# A Twenty-Year Study of a Single Institution Examining Age, Gender, and Demographic Differences Between Subcapital and Peritrochanteric Hip Fractures

**DOI:** 10.7759/cureus.78528

**Published:** 2025-02-04

**Authors:** Fanouria Pinelopi Margariti, Eustathios Kenanidis, Panagiotis Konstantinos Emfietzis, Olga Sitsiani, Eleftherios Tsiridis

**Affiliations:** 1 Academic Orthopaedic Department, Papageorgiou General Hospital, Aristotle University of Thessaloniki, School of Medicine, Thessaloniki, GRC

**Keywords:** femoral neck fractures, hip fractures, intertrochanteric hip fractures, peritrochanteric fractures, subcapital hip fractures

## Abstract

Introduction: Limited data indicate demographic differences between peritrochanteric and subcapital hip fractures. This study aimed to document the 20-year hospitalisation rates for hip fractures at a tertiary hospital and compare the prevalence and demographics between the two types of hip fractures.

Materials and methods: This retrospective study examined the electronic records of patients admitted for hip fractures at our hospital from January 2004 to August 2023, utilising the 10th revision of the International Classification of Diseases codes. We collected demographic data encompassing age, sex, and fracture characteristics.

Results: A total of 5,987 hip fractures (55.4% peritrochanteric and 44.6% subcapital) were included. The number of patients admitted with hip fractures decreased during our country's economic crisis and the COVID-19 pandemic. The incidence of subcapital and peritrochanteric hip fractures over the two decades was comparable (p = 0.232). There were two-and-a-half times more female cases than male cases. Peritrochanteric fractures were more common than subcapital fractures in both sexes, but the ratio between the sexes was comparable (p = 0.532). A significant difference was observed in the mean age (years) of women (80.4 ± 10.1) compared to men (77.0 ± 14.1) (p < 0.001) and between subcapital (77.8 ± 11.9) and peritrochanteric hip fractures (80.7 ± 10.9) (p < 0.001). The mean age of women with peritrochanteric fractures (81.9 ± 8.9) was statistically different from those with subcapital hip fractures (78.4 ± 11.0) (p < 0.001). This difference was not significant for men (p = 0.114). Individuals over 75 years old were involved in 76.8% of all cases, with the majority being women (57.5%). There was a significant increase in the ratio of female to male cases over 75 years (p < 0.001).

Conclusions: Our research indicated that women are more likely to develop hip fractures than men. Additionally, peritrochanteric fractures are more common than subcapital fractures. The risk of developing hip fractures increases with age, particularly after 75. Furthermore, hip fractures in women occur significantly later than in men, and the same applies to peritrochanteric compared to subcapital fractures. There may be demographic disparities between peritrochanteric and subcapital hip fractures.

## Introduction

Hip fractures are among the most serious complications of osteoporosis. In Europe, they rank as the second most common type of osteoporotic fracture, following vertebral fractures [1]. Hip fractures pose a major health concern for older adults, resulting in significant morbidity, mortality, and healthcare costs [1]. As life expectancy increases, the incidence of hip fractures also increases. By 2050, the annual number of hip fractures is predicted to reach 6.26 million [2]. Addressing the hip fracture epidemic is essential.

Hip fractures have traditionally been classified into intracapsular and extracapsular fractures [3]. Intracapsular fractures primarily involve subcapital fractures, whereas extracapsular (or peritrochanteric) fractures include intertrochanteric and subtrochanteric fractures [3-5]. Intertrochanteric fractures occur between the greater and lesser trochanters, while subtrochanteric fractures occur in the subtrochanteric region [3-5].

Limited published data suggest that there may be demographic differences between patients with extracapsular and intracapsular hip fractures [3,4]. Some studies have found that elderly individuals with osteoporosis are at a higher risk of experiencing extracapsular fractures compared to intracapsular fractures [5]. However, another study has shown conflicting results [6]. Furthermore, there is limited information on how gender influences falls and hip fractures among older individuals. Studies have demonstrated that older women tend to experience more intertrochanteric fractures, while younger men are more prone to subcapital fractures [3,4,7-10]. It has been reported that intertrochanteric fractures are more closely related to osteoporosis than subcapital fractures [4,11].

The incidence rate of hip fractures in Greece remains unclear due to a scarcity of studies, complicating the task of determining the scale of the issue by age and gender. This study aimed to record the number of hip fractures, both intra- and extracapsular, admitted to a tertiary hospital over the last 20 years and to identify demographic differences among them. Specifically, it sought to analyse variations in the prevalence of hip fractures across the two decades and to assess how age and gender influenced the ratio of intracapsular to extracapsular fractures. Differences in age and sex may offer important insights into trends, risk factors, and local healthcare requirements, guiding clinical strategies, preventive measures, and public health policies.

## Materials and methods

Study design and patient selection

This retrospective cohort study was conducted in the academic orthopaedic department of Papageorgiou General Hospital and received ethical approval from the Institutional Research Board. All patient information was de-identified, and patient consent was not required. Data collection commenced in August 2023. Alongside three other hospitals, ours serves a population of approximately 2,000,000 individuals in a mix of rural, urban, and suburban areas in our country. The hospital has a total capacity of 750 beds.

The study included patients with hip fractures admitted to our hospital between January 2004 and August 2023. The inclusion criteria consisted of patients with (1) no age restriction, (2) unilateral extracapsular (peritrochanteric including intertrochanteric and subtrochanteric) and intracapsular (subcapital) hip fractures, and (3) those treated either surgically or conservatively for hip fractures. The exclusion criteria included patients with (1) bilateral hip fractures, (2) polytrauma, (3) periprosthetic or pathological fractures, and (4) incomplete medical information (lack of demographics or type of fracture). 

Data collection

This retrospective study examined the records of patients admitted for hip fractures at the Orthopaedic Departments of our General Hospital between January 2004 and August 2023. The patients were identified using the International Classification of Diseases 10 (ICD-10) diagnostic codes within our hospital's electronic record database. We retrieved all cases with ICD-10 codes S72.0 (subcapital fractures) and S72.1 (peritrochanteric fractures). We collected demographic data, including age, sex, and other characteristics of fractures, for patients admitted to our hospital with these two ICD-10 codes during the last two decades. 

The sample was examined as a whole; however, it was further divided into two age groups, those under 75 years old and those over 75, for additional analysis. Over the past two decades, we have documented the number of patients admitted to our hospital with hip fractures and compared the prevalence of these fractures between the two decades. Furthermore, we aimed to identify the differences in demographics (gender and age) between the two most common types of hip fractures: subcapital and peritrochanteric fractures.

Statistical analysis

Standard statistical methods were used for descriptive statistics. The normality of data distribution was assessed using the Kolmogorov-Smirnov and Shapiro-Wilk tests. Two-tailed statistical tests were used. The p-values <0.05 were considered statistically significant. A two-sided independent-sample t-test was used to compare continuous variables normally distributed, and the chi-square test (x^2^ test) was used to compare categorical variables. Statistical analyses were performed using IBM SPSS Statistics for Windows, version 27.0, released in 2020 (IBM Corp, Armonk, NY).

## Results

**Figure 1:**
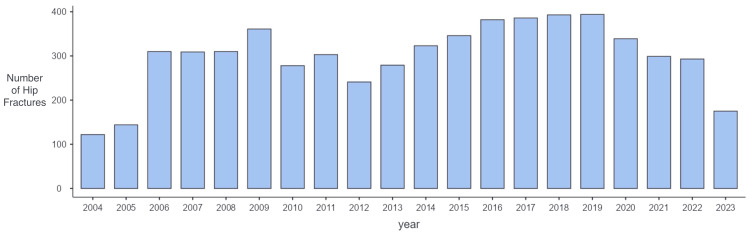
The annual incidence of hip fractures admitted to the hospital (2004-2023).

A total of 5,987 hip fracture cases that met the inclusion criteria were identified during the study period and finally included. Among all the hip fracture cases, 3,315 (55.4%) were peritrochanteric fractures, while 2,672 (44.6%) were subcapital fractures. In 2019, there were 394 hip fractures, the highest occurrence recorded. There was a decline in the annual admission of patients with hip fractures from 2010 to 2012 and from 2020 to 2022. The number of patients with hip fractures admitted to the hospital each year is illustrated in Figure 1.

Between 2004 and 2013, 2,657 patients with hip fractures were admitted to the hospital. Of these, 1,163 cases were subcapital, while 1,494 were peritrochanteric hip fractures. In the second decade, from 2014 to 2023, the hospital admitted 3,330 patients with hip fractures, including 1,509 with subcapital fractures and 1,821 with peritrochanteric fractures. No significant difference was observed in subcapital and peritrochanteric hip fracture incidence between the two decades (x^2 ^test, p = 0.232). 

**Figure 2:**
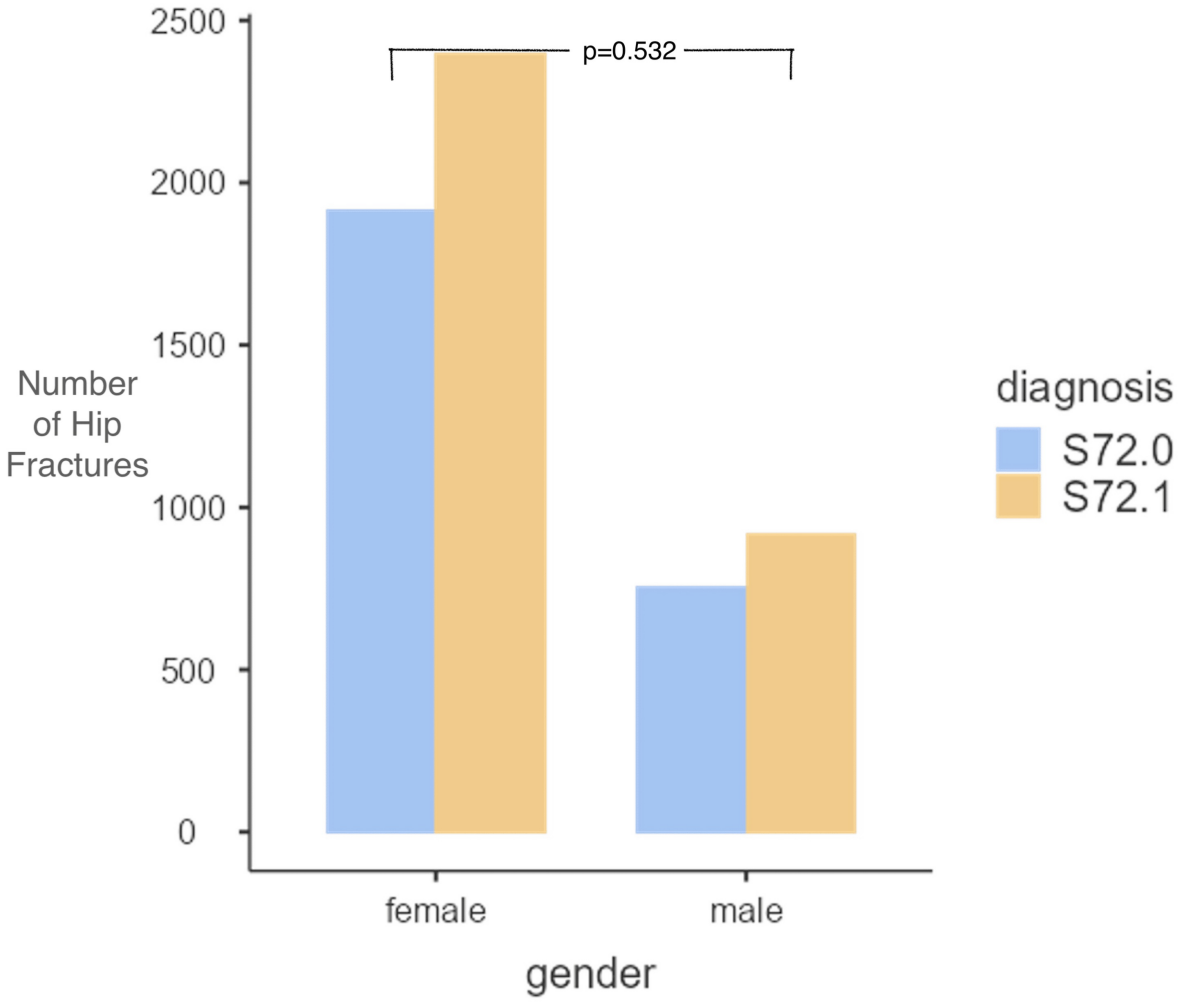
The number of male and female patients admitted to the hospital with ICD-10 diagnosis S72.0: subcapital and S72.1: peritrochanteric femoral hip fracture. The ratio of peritrochanteric to subcapital hip fractures was not significantly different between male and female patients. Test was performed using the x^2^ test. ICD: International Classification of Diseases.

Hip fractures were more prevalent in women, with 4,315 (72.1%) cases reported compared to 1,672 (27.9%) in men, indicating a rate that is 2.5 times higher. Peritrochanteric fractures occurred more frequently than subcapital fractures in both genders. Among women, there were 2,400 cases (40.1%) of peritrochanteric fractures, while 1,915 cases (32%) were classified as subcapital fractures. In men, 915 cases (15.3%) were peritrochanteric fractures, against 757 cases (12.6%) of subcapital fractures. The ratio of peritrochanteric to subcapital hip fractures did not show a significant difference between male and female cases (x^2^ test, p = 0.532) (Figure 2).

**Figure 3:**
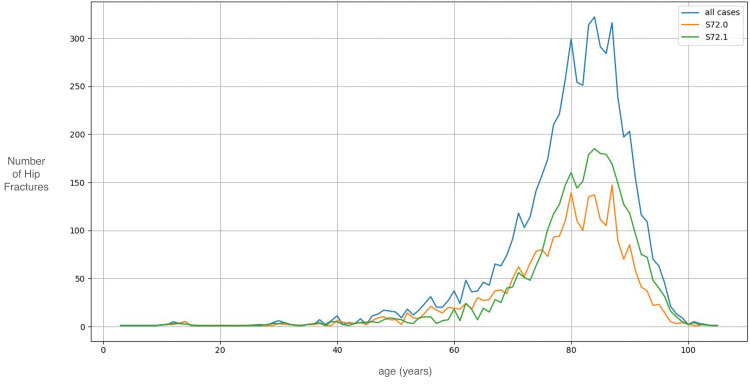
The distribution of hip fractures, classified into peritrochanteric (ICD-10 code: S72.1) and subcapital (ICD-10 code: S72.0) categories admitted to our hospital in comparison with patients' age. ICD: International Classification of Diseases.

The mean age of patients admitted with a hip fracture to our hospital over the last 20 years was 79.4 ± 11.4 years. Figure 3 displays the number of hip fractures categorised as peritrochanteric and subcapital admitted to our hospital by age for the whole period.

**Figure 4:**
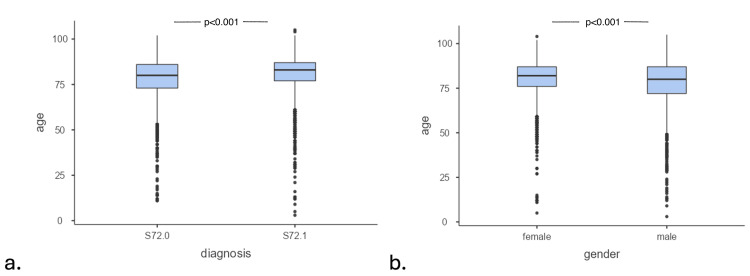
The box plots illustrate the distribution of ages among patients admitted to the hospital with hip fractures, specifically in relation to a. type of fracture (ICD-10 code:S72: subcapital, S72.1: peritrochanteric) and b. gender. The mean age was significantly different between a. subcapital and peritrochanteric fractures and b. between men and women. Tests were performed using the Student's t-test. ICD: International Classification of Diseases.

The mean age ± standard deviation (SD) of female patients admitted for hip fracture was 80.4 ± 10.1 years, while for male patients, it was 77.0 ± 14.1 years. A significant difference in the mean age between men and women was observed (Student's t-test, p < 0.001). The mean age (±SD) of patients who experienced a subcapital fracture was 77.86 years (±11.9), while the mean age (±SD) of patients with a peritrochanteric fracture was 80.7 years (±10.9). The nearly three-year difference between the mean age of subcapital and peritrochanteric hip fractures was statistically significant (Student's t-test, p < 0.001) (Figure 4).

The mean age (±SD) for female cases with peritrochanteric fractures was 81.9 years (±8.92), while for those with subcapital fractures, it was lower at 78.40 years (±11.00). This difference was statistically significant (Student's t-test, p < 0.001). The mean age (±SD) for male cases with peritrochanteric fractures was 77.5 years (±14.5), whereas for those with subcapital fractures, it was lower at 76.4 years (±13.7); this difference was not significant (Student's t-test, p = 0.114).

Out of 4,600 hip fracture cases, 76.8% were individuals aged 75 years or older, while 1,387 cases, representing 23.2%, were younger than 75 years. Among those over 75 years, there were 3,443 female cases (57.5%) and 1,157 male cases (19.3%). For the younger group, there were 872 female cases (14.6%) and 515 male cases (8.6%). The ratio of male-to-female cases differed significantly before and after the age of 75 (x^2^ test, p < 0.001). Female cases outnumbered male cases before and after age 75, with the increase in female cases being notably higher after age 75.

Beyond the age of 75, 2,713 (45.3%) hip fractures were peritrochanteric, while 1,887 (31.5%) were subcapital. Before the age of 75, 602 (10.1%) hip fractures were peritrochanteric and 785 (13.1%) were subcapital. The subcapital-to-peritrochanteric hip fracture ratios differed significantly before and after the age of 75 (x^2^ test, p < 0.001). A notable rise in the number of peritrochanteric hip fractures was observed in patients aged 75 years and above.

## Discussion

Our retrospective cohort study demonstrated that the number of hip fractures admitted to this tertiary general hospital remained constant in the previous two decades but decreased during the economic crisis and the COVID-19 pandemic in our country. During this period, women had a 2.5 times higher risk of hip fractures compared to men. Peritrochanteric fractures were also more prevalent than subcapital hip fractures. The average age of women who suffered a hip fracture was three years older than that of men. The mean age of patients sustaining a subcapital fracture was three years younger than those with peritrochanteric fractures. There has been a significant increase in the number of hip fractures occurring in individuals over the age of 75, particularly among women who experience peritrochanteric fractures. As women age, the incidence of extracapsular fractures rises, whereas in men, there is a slight decline in such fractures with increasing age groups.

Hip fractures are associated with high mortality rates and a substantial economic burden due to patient characteristics, surgical procedures, and hospital-level factors [12]. The costs related to hip fractures surpass those for breast and gynaecological cancers combined, highlighting the necessity for further exploration into prevention and treatment [12]. It is projected that the total number of hip fractures will reach 6.29 million by 2025, with the incidence being twice as high in women as in men, according to the International Osteoporosis Foundation [12]. Our study revealed that women were 2.5 times more likely to suffer hip fractures than men, which is consistent with other published data [13,14]. Moreover, individuals aged over 75 years faced a higher risk of sustaining a hip fracture. As we age, our bone density gradually diminishes, heightening the risk of osteoporosis. Consequently, elderly individuals are more vulnerable to severe osteoporosis and have an increased likelihood of suffering falls, which can lead to hip fractures [15]. Studies have shown that the rate of hip fractures doubles with every decade of life after the age of 50. In the UK, the 10-year risk of hip fracture for women increases from 0.3% at age 50 to 8.7% at age 80 [16]. Besides, as individuals age, their risk of falling rises due to diminished physical and cognitive abilities. Difficulties in walking and loss of balance are common causes of falls among the elderly [17]. Studies have demonstrated that older women are at a higher risk of falling, which significantly contributes to their increased likelihood of hip fractures [18]. The accelerated deterioration of female muscles with advancing age, a sedentary lifestyle, poor nutrition, and diminished postural control during stressful balance situations may elucidate the disparities in hip fractures between genders [19]. Socioeconomic factors and mental health issues, such as depression and the use of multiple medications, are also recognised risk factors for falls [19]. Although men are less likely to experience hip fractures compared to women, they tend to suffer fractures at a younger age due to injuries incurred during physical activities rather than from age-related factors [19].

Research indicates that fall prevention can significantly reduce hip fractures in older adults, as falls are a considerable risk factor alongside osteoporosis [20]. Factors such as loss of muscle mass, impaired coordination, and gait disturbances due to pain are primary reasons for reduced physical activity among the elderly. A prospective cohort study suggests that these issues can heighten their susceptibility to falls and fall-related fractures [21]. Older individuals are advised to participate in physical activities to improve their quality of life, enhance bone density and muscle mass, and avert falls and associated fractures. The nature of a hip fracture may be determined by the angle at which the greater trochanter impacts the floor [22]. Literature indicates that the most prevalent cause of hip fractures in older adults is a sideways fall resulting in lateral hip impact [23]. Another significant cause of hip fracture is an injury sustained from knee impact, which delivers sufficient energy to the proximal femur through axial loading [24]. Changes in the characteristics of falls among older adults over recent decades may have contributed to a rise in peritrochanteric hip fractures, particularly in individuals over 75.

Our study found that peritrochanteric hip fractures increased significantly with age in women, while no such trend was observed in men. The results are consistent with existing literature showing a continuous rise in extracapsular hip fractures in women compared to intracapsular ones [13,25]. Studies conducted in North America [26] and Norway [25] have demonstrated that the incidence of extracapsular hip fractures in women increases with age at a similar rate. The higher risk of hip fractures in women may be attributed to their increased susceptibility to osteoporosis, as well as their longer life expectancy, particularly in Western populations [27]. Evidence suggests that bone loss patterns may differ between sexes, and certain morphometric and densitometric features of the femoral neck may predict the type of hip fracture [28]. Research has shown that women with a femoral neck bone density equal to or greater than 1.0 g/cm^2^ are less likely to experience hip fractures. On the other hand, patients with an intertrochanteric bone density of less than 0.6 g/cm^2^ are twice as likely to experience extracapsular hip fractures [28].

Our study suggests that the decrease in hip fractures during the COVID-19 quarantine and the economic crisis in our country may be linked to falls. Several previous studies demonstrated a significant decline in surgically treated fractures, particularly high-energy fractures, and a slight reduction in the incidence of hip fractures during the COVID-19 pandemic [29]. Strict quarantine measures were implemented during the COVID-19 outbreak, including a ban on leaving the house. Widespread fear was propagated through the media. Consequently, visiting places that could increase the risk of falls and hip fractures was discouraged among older individuals. Bud et al. reported a 29.9% decrease in cases after introducing pandemic measures. They observed a change in the time patients took to seek medical help following an injury. However, these cases had no overall complication increase [30].

This retrospective cohort study has certain limitations. First, the sample is not geographically representative of the entire country; however, it is considered representative of the city’s Caucasian population. The main advantage of our study is that it utilises a 20-year continuous record of hip fracture admissions to a tertiary general hospital in our country's second-largest city. Secondly, no sample size estimation has been conducted for this study. The study's retrospective approach and the exclusive use of ICD-10 coding for the electronic database search are further limitations to our research, restricting our ability to identify lost cases. Furthermore, changes in healthcare access and diagnostic practices over the past 20 years hinder the full applicability of our findings.

## Conclusions

Our research demonstrated a similar incidence of subcapital and peritrochanteric hip fractures over the last two decades and revealed demographic disparities between these types of fractures. Women were more likely to develop hip fractures than men, and peritrochanteric fractures were also more common than subcapital ones. The risk of developing hip fractures increases with age, particularly after 75 years. Furthermore, hip fractures in women occurred significantly later than in men. A similar pattern was observed for peritrochanteric fractures compared to subcapital fractures. Understanding the complex causes and mechanisms of hip fractures is crucial for developing effective preventive interventions and improving the health outcomes of various elderly populations.
